# Retrospective Comparison of Conventional Triple Therapy and Levofloxacin-Based Sequential Therapy for Helicobacter pylori Eradication in Mardan Medical Complex

**DOI:** 10.7759/cureus.98803

**Published:** 2025-12-09

**Authors:** M Naeem, Manzoor Hussain

**Affiliations:** 1 Gastroenterology, Mardan Medical Complex, Mardan, PAK; 2 General Medicine, Mardan Medical Complex, Mardan, PAK

**Keywords:** antibiotic resistance, dyspepsia, eradication rate, helicobacter pylori, levofloxacin, proton pump inhibitor, sequential therapy, triple therapy

## Abstract

Objective: This study aims to compare, using record-based data, the clearance proportions of the conventional triple management regimen and the levofloxacin-based sequential management regimen in patients treated at Mardan Medical Complex.

Methods: A record-based review was conducted from January to June 2024 among adults aged 18 to 65 years with stool antigen-confirmed *Helicobacter pylori* infection. Data were collected for demographics, management regimens, and post-management stool antigen results. Statistical analyses included Chi-square and independent t-tests (p < 0.05 notable).

Results: Among 238 patients, clearance was obtained in 62% of those receiving the triple management regimen and 80% of those receiving the sequential management regimen (χ² = 9.03, p = 0.003). The sequential management regimen showed greater clearance and better tolerance.

Conclusion: Sequential management regimen obtained superior clearance proportions and should be preferred in areas with high clarithromycin resistance.

## Introduction

*Helicobacter pylori* is a Gram-negative bacterium that colonizes the human gastric mucosa and is a key factor in gastritis, peptic ulcer disease, and gastric carcinoma [1,2]. Globally, more than half of the population is infected, with greater prevalence in developing regions due to poor sanitation and overcrowding [3]. For several decades, the standard first-line management has been a clarithromycin-based triple management regimen comprising a proton pump inhibitor (PPI), clarithromycin, and amoxicillin [4]. However, the increasing resistance to clarithromycin has notably reduced clearance success proportions [5]. Levofloxacin-based sequential management regimen introduces antibiotic rotation within the same management course, potentially overcoming resistance mechanisms [6,7]. Given the limited regional data in Pakistan, this record-based analysis aimed to assess and compare the clearance proportions of triple and sequential regimens in real-world clinical practice. *H. pylori* colonization remains a notable global public health issue, contributing to chronic gastritis, peptic ulcer disease, and gastric malignancies. The rise in antimicrobial resistance, particularly to clarithromycin and metronidazole, has compromised the efficacy of standard regimens. Sequential regimens introduce a two-phase approach by varying antibiotic exposure during the same treatment course, which may help overcome resistance mechanisms and improve eradication rates. However, regional data on comparative effectiveness remain limited, emphasizing the need for localized studies like the present one. Global data have shown resistance rates rising across multiple regions, underscoring the need for alternative regimens [8,9].

## Materials and methods

Study design

A record-based observational analysis was conducted at the Department of Medicine, Mardan Medical Complex, and Bacha Khan Medical College, Mardan, Pakistan.

Study Period

The study was conducted from January to June 2024.

Geographical context

Mardan is a major city in Khyber Pakhtunkhwa, Pakistan, serving as a referral center for gastroenterological diseases across the region.

Participants

Medical records of adults aged 18 to 65 years diagnosed with stool antigen-confirmed *H. pylori* colonization were reviewed. Sampling was non-probability consecutive, including all eligible patients treated during the analysis period.

Inclusion criteria

Inclusion criteria included patients aged 18 to 65 years with positive stool antigen tests who completed the prescribed management regimen.

Exclusion criteria

Exclusion criteria included patients with incomplete records, previous eradication management regimen, pregnancy, recent antibiotic use (within 4 weeks), or severe systemic illness.

Treatment groups

Treatment groups included Group A (Triple Therapy): clarithromycin 500 mg, amoxicillin 1 g, and PPI twice daily for 14 days; and Group B (Sequential Therapy): amoxicillin 1 g and PPI for five days, followed by levofloxacin 500 mg and PPI for five days.

Outcome measure

Eradication was confirmed by stool antigen test four weeks post-management.

Data analysis

Data were analyzed using IBM SPSS Statistics for Windows, Version 26 (Released 2018; IBM Corp., Armonk, New York). Mean ± SD and interquartile ranges (IQR) were calculated for continuous variables. Frequencies and percentages were used for categorical data. The independent t-test and chi-square test were applied, with significance set at p < 0.05. The independent t-test was used to compare mean age between treatment groups, while the chi-square test was used for categorical variables such as gender and eradication rates.

## Results

A total of 238 eligible patients were analyzed, with 119 patients assigned to the triple therapy group (Group A) and 119 patients assigned to the sequential therapy group (Group B). The mean age was 41.2 ± 12.3 years (IQR: 32-50) in Group A and 42.5 ± 11.7 years (IQR: 34-51) in Group B. Gender distribution was similar between groups (68 males/51 females in Group A; 65 males/54 females in Group B; p = 0.68), indicating baseline comparability (see Table 1).

**Table 1:** Baseline Demographic and Clinical Characteristics No statistically significant difference was found between groups for age or gender distribution (p > 0.05).

Variable	Triple Therapy (n = 119)	Sequential Therapy (n = 119)	Test Used	p-value
Mean age (years)	41.2 ± 12.3 (IQR 32–50)	42.5 ± 11.7 (IQR 34–51)	Independent t-test = 0.83	0.41
Gender (M : F)	68 : 51	65 : 54	—	0.68

The overall eradication rate for the triple therapy regimen was 62% (74/119), while for the sequential therapy regimen it was 80% (95/119). This difference was statistically significant (χ² = 9.03, p = 0.003). The sequential regimen was not only more effective but also better tolerated, with fewer recorded complaints of nausea or abdominal discomfort (as shown in Table 2).

**Table 2:** Treatment Outcomes and Adverse Effects The sequential management regimen demonstrated greater clearance proportions (p = 0.003) compared to the triple management regimen. While fewer adverse effects were reported in the sequential group, the difference was not statistically significant (p = 0.12).

Outcome	Triple Therapy (n = 119)	Sequential Therapy (n = 119)	Test Used	p-value
Eradication rate (%)	62%	80%	χ² = 9.03	0.003
Reported side effects (%)	12%	6%	Chi-square	0.12

The independent t-test analysis showed no statistically significant difference in mean age between the two treatment groups (t = 0.83, p = 0.41). This confirms that both cohorts were comparable in baseline demographics. Likewise, gender distribution did not differ significantly (p = 0.68), supporting the validity of subsequent outcome comparisons.

Baseline comparability between groups was verified through the independent t-test and chi-square analyses, ensuring that outcome differences were not influenced by demographic imbalance.

## Discussion

This record-based analysis demonstrated that the levofloxacin-based sequential therapy achieved significantly higher eradication rates than the conventional clarithromycin-based triple therapy, consistent with findings from multiple international studies [7,10-12]. The improvement in eradication outcomes reflects the growing evidence supporting sequential regimens in areas where antibiotic resistance has reduced the efficacy of traditional approaches [5,8,9]. These results reinforce that sequential therapy offers a superior clinical alternative in regions with high clarithromycin resistance.

Several mechanisms may explain this improved efficacy. The initial amoxicillin-PPI phase disrupts bacterial cell wall integrity, enhancing the penetration and activity of subsequent antibiotics such as levofloxacin [11,13]. Furthermore, levofloxacin has excellent gastric mucosal penetration and broad-spectrum antibacterial coverage, making it a valuable substitute in regions where resistance to clarithromycin is prevalent [4,6]. These pharmacological advantages contribute to the superior eradication performance observed in this study and are consistent with previously published data [10,12].

Our findings align with those of regional and international studies conducted in Pakistan, Asia, and Europe, where triple therapy success rates have declined below the acceptable threshold of 80% [8-10]. Sequential therapy provides a rational and practical alternative that can be implemented without major changes to clinical infrastructure. By incorporating antibiotic rotation within a single course, sequential therapy can help mitigate resistance development, particularly in resource-limited settings where culture-based susceptibility testing is not routinely available [5,13].

Baseline comparability between groups was confirmed through independent t-test and chi-square analyses, which ruled out potential demographic bias. This strengthens the internal validity of our results and supports the conclusion that treatment differences were due to the regimens themselves rather than variations in patient characteristics. The statistical significance observed (p = 0.003) indicates a true difference in therapeutic outcomes rather than random variation.

Clinically, these results underscore the importance of adopting region-specific treatment strategies. As antibiotic resistance patterns vary geographically, national treatment guidelines should prioritize regimens that reflect local susceptibility profiles. Sequential therapy offers a flexible, cost-effective, and evidence-based option that may enhance eradication rates and improve patient adherence due to better tolerability.

Nevertheless, the study has limitations. As a retrospective single-center analysis, it cannot account for all potential confounding factors. Adherence was inferred from clinical documentation rather than direct observation, and bacterial culture or molecular resistance profiling was not available. Despite these limitations, the consistent documentation and the size of the patient cohort lend strength to the findings. Future multicenter prospective trials with microbiological confirmation of resistance would provide deeper insights into the evolving efficacy of sequential and levofloxacin-based regimens.

In summary, sequential therapy demonstrated superior eradication outcomes and tolerability compared with standard triple therapy. Its integration into clinical practice should be considered in regions with established clarithromycin resistance, while continuous surveillance and antimicrobial stewardship remain essential to sustain long-term treatment success. Comparable outcomes were observed in meta-analyses and regional reports highlighting the growing clinical role of sequential regimens [10-12].

Limitations

This single-center record-based analysis may not represent the entire regional population. Compliance was inferred from clinical records rather than direct observation. Resistance profiling through culture or molecular testing was not performed, which could have provided mechanistic insights.

## Conclusions

Levofloxacin-based sequential therapy achieved higher eradication rates and better tolerability compared with conventional clarithromycin-based triple therapy. These results emphasize the importance of adopting sequential management as a preferred first-line regimen in areas where clarithromycin resistance is common. Implementation of antibiotic stewardship and local resistance monitoring programs can help optimize clearance proportion. Further multicenter randomized trials are recommended to validate these findings and guide regional management guidelines.
